# Interplay: The Essential Role between INSM1 and N-Myc in Aggressive Neuroblastoma

**DOI:** 10.3390/biology11101376

**Published:** 2022-09-20

**Authors:** Chiachen Chen, Michael S. Lan

**Affiliations:** Department of Genetics, Louisiana State University Health Sciences Center, New Orleans, LA 70112, USA

**Keywords:** INSM1, *MYCN*, sympathoadrenal lineage, neuroblastoma, neuroendocrine differentiation

## Abstract

**Simple Summary:**

Neuroblastoma (NB) is a cancer that starts in certain very early forms of nerve cells of the sympathetic nervous system, most often found in an embryo or fetus. Symptoms may include bone pain, an abdominal mass, frequent urination, limping, anemia, spinal cord weakness, or bruising of the eye area. N-Myc is a key driver of high-risk NB. An elevated expression of N-Myc often predicts a poorer prognosis, in both time to tumor progression and overall survival rate. We discovered a transcription factor, *insulinoma-associated-1* (*INSM1)*, as the downstream target gene of N-Myc. INSM1 has emerged as a novel NB biomarker that plays a critical role in facilitating NB tumor cell development. Both N-Myc and INSM1 demonstrate high clinical relevance to NB. Therefore, further understanding the association of INSM1 and N-Myc functions in aggressive NB should be beneficial for future NB treatment.

**Abstract:**

An aggressive form of neuroblastoma (NB), a malignant childhood cancer derived from granule neuron precursors and sympathoadrenal lineage, frequently comprises *MYCN* amplification/elevated N-Myc expression, which contributes to the development of neural crest-derived embryonal malignancy. N-Myc is an oncogenic driver in NB. Persistent N-Myc expression during the maturation of SA precursor cells can cause blockage of the apoptosis and induce abnormal proliferation, resulting in NB development. An insulinoma-associated-1 (INSM1) zinc-finger transcription factor has emerged as an NB biomarker that plays a critical role in facilitating tumor cell growth and transformation. INSM1 plays an essential role in sympathoadrenal cell differentiation. N-Myc activates endogenous INSM1 through an *E2-box* of the *INSM1* proximal promoter, whereas INSM1 enhances N-Myc stability via RAC-α-serine/threonine protein kinase (AKT) phosphorylation in NB. The ectopic expression of INSM1 stimulates NB tumor growth in contrast to the knockdown of INSM1 that inhibits NB cell proliferation. The clinical pathological result and bioinformatics analysis show that INSM1 is a strong diagnostic and a prognostic biomarker for the evaluation of NB progression. The INSM1/N-Myc expression shows high clinical relevance in NB. Therefore, targeting the INSM1/N-Myc-associated signaling axis should be a feasible approach to identifying new drugs for the suppression of NB tumor growth.

## 1. Introduction

Neuroblastoma (NB) is a cancer that starts in early nerve cells (called neuroblasts) of the sympathetic nervous system, so they can be found anywhere along this system. Most NB begins in the abdomen, either in an adrenal gland or in the sympathetic nerve ganglia. The rest starts in the sympathetic ganglia near the spine, in the chest, in the neck, or in the pelvis. Human NB is the most common childhood extracranial tumor arising from the sympathetic nervous system. It is also a clinically heterogeneous disease that ranges from spontaneous regression to high-risk stage 4 disease. This type of cancer occurs most often in infants and young children. NB accounts for about 6–7% of all cancers in children as the most common cancer in babies and the third-most common cancer in children after leukemia (26%) and brain cancer (21%). Although *MYCN* was identified as an important genetic biomarker in high-risk NB, it was not considered “druggable” in the standard care of *MYCN*-amplified NB patients. However, in high-risk NB, the survival rate is about 40%, with a long-term survival rate of only 15%. A successful therapeutic strategy for high-risk NB patients is urgently needed and represents an unmet societal necessity.

Recent studies of a neuroendocrine (NE) transcription factor, Insulinoma-associated-1 (INSM1), revealed that this zinc-finger DNA-binding protein not only regulates downstream target genes but also modulates signaling pathways critical for the stability of the N-Myc oncogenic protein [1]. By screening a small molecule library using *INSM1* promoter-driven luciferase assay, we were able to identify multiple inhibitors potently inhibiting *INSM1* promoter activity as well as NB tumor cell growth [2]. The down regulation of INSM1 also inhibits N-Myc protein expression, which contributes to NB tumor suppression [3]. When we dissect the transcriptional regulation of INSM1 and N-Myc functional roles in NB, it is plausible to reveal potential small molecules targeting both INSM1 and N-Myc. In the current review article, we discuss the essential role of INSM1 and N-Myc interplay in aggressive NB as potential targets for NB cancer therapy.

### 1.1. INSM1 Is a Unique Zinc-Finger Transcription Factor

Insulinoma-associated-1 (INSM1) is a zinc-finger transcription factor that was originally identified in a human insulinoma subtraction library [4]. *INSM1* is an intronless gene located in chromosome *20 p11.2*, encoding a 510-amino-acid polypeptide consisting of a Snail/Gfi (SNAG) domain, two proline-rich segments, four dibasic amino acid sites, a putative nuclear localization signaling sequence (NSL), and a potential amidation signal sequence (PGKR) in the N-terminus. The C-terminal portion of INSM1 contains five zinc-finger motifs, which constitute a functional domain of a DNA-binding transcription factor. The DNA-binding consensus sequence of INSM1 was determined by a selected and amplified random oligonucleotide binding assay as 5′-T^G^/_T_^C^/_T_^C^/_T_^T^/_A_GGGG^G^/_T_C^G^/A-3′ [5]. Several downstream target genes’ promoters of INSM1 were subsequently identified based upon the consensus sequence on the promoter regions including neurogenic differentiation factor (NeuroD1), insulin, RE1 silencing transcription factor (REST), ripply transcription repressor 3 (Ripply3), neurogenin 3 (Ngn3), adherens junction belt-specific protein (Plekha7), and INSM1 itself [5,6,7]. SNAG domain functions as a transcription repressor by recruiting the histone deacetylase Hdac1, Hdac2, Rcor1, and histone demethylase Kdmla to modulate gene expression by chromatin modification [8,9,10]. INSM1 protein not only binds to DNA as a transcription factor but also directly binds to cellular proteins via its proline-rich domain. INSM1 is capable of binding to cyclinD1 or RACK1 directly to interfere with cell signaling and cell cycle progression [11,12].

The expression of INSM1 was detected in early human fetal brain, endocrine pancreas development, and the tumors of NE origin including insulinoma, retinoblastoma, neuroblastoma, medulloblastoma, pheochromocytoma, Merkel cell carcinoma, medullary thyroid carcinoma, pituitary tumor, carcinoids, and small-cell lung carcinoma [1]. The upstream region of the *INSM1* promoter (2090 bps) contains several tissue-specific regulatory elements that might contribute to a distinctive expression pattern of INSM1 dominant in tumors of NE origin, as well as displays its role in neurogenesis and NE cell differentiation during embryonic development [5]. During embryonic development, the expression of INSM1 was dramatically reduced in a later stage and mostly silenced in normal adult tissues. The transient expression pattern suggests that the *INSM1* gene functions as a developmentally regulated transcription factor, which transiently expresses in early NE cells and is highly reactivated in NE tumors.

### 1.2. The Role of INSM1 in Sympathoadrenal Cell Differentiation

**Figure 1:**
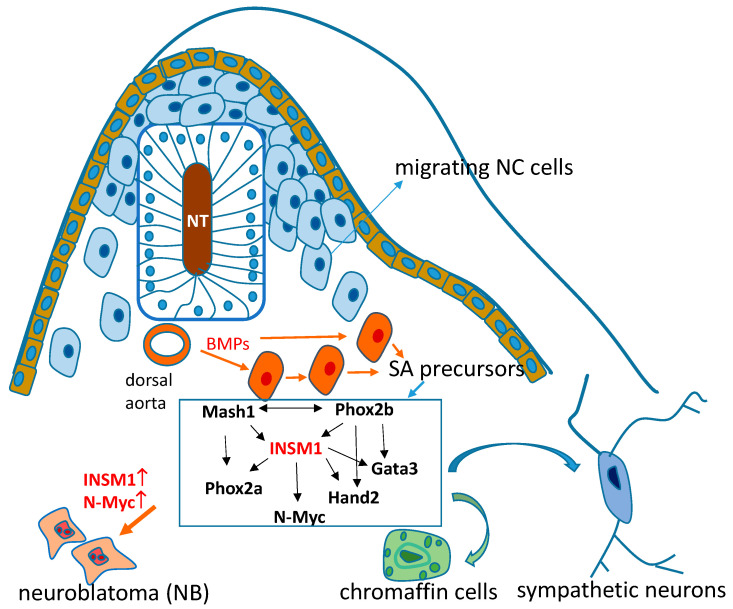
Role of INSM1 in SA lineage differentiation. Migrating neural crest (NC) cells from neural tube (NT) aggregated and stimulated by bone morphogenic proteins (BMPs) from the dorsal aorta wall for symphtho-adrenal (SA) progenitor cell differentiation. A common trans-regulatory network of specific transcription factors include Phox2b, Phox2a, Mash1 (Ascl-1), Hand2, Gata2/3, INSM1, and N-Myc, SA precursors further differentiate into sympathetic neurons and chromaffin cells, or NB tumor formation.

The neural crest (NC) is a transient structure during early embryonic development. NC cells from the trunk area of the neural tube generate a population of multipotent embryonic cells. The sympathoadrenal (SA) cells’ lineage is a major sub-lineage of the NC cells that further differentiate into sympathetic neurons, chromaffin, and intermediate small intensely fluorescent (SIF) cells. Sympathetic neurons and chromaffin cells are derived from a common SA progenitor, which develops from NC cells that aggregated the dorsal aorta [13,14,15]. Bone morphogenic proteins (BMPs) secreted from the dorsal aorta wall play a central role in the differentiation of SA progenitor cells [16,17]. The studies of chick embryo models showed that the expression of BMPs in the wall of the dorsal aorta attracts NC cells expressing BMP-receptor IA and BMP-receptor IB migrating into the dorsal aorta area [18]. BMPs induce the expression of SA precursor-specific markers. The inhibition of BMP4/7 abolished the expression of catecholaminergic and neuronal markers in NC cells that had aggregated at the dorsal aorta [19,20,21]. These data demonstrate that, at least in the chick embryo, BMPs are required in vivo for the development of SA cells. The differentiation of the SA lineage from the SA progenitor cells now aggregated in the dorsal aorta area is under the control of a common trans-regulatory network of specific transcription factors including Phox2b, Phox2a, Ascl-1, Hand2, Gata2/3, N-Myc, and INSM1 (Figure 1). All components of the SA transcriptional network have been attributed to different functions in various parts of SA cell development, including the promotion of noradrenergic, neural, and endocrine cell differentiation; the regulation of proliferation; and cell apoptosis [13].

INSM1 plays an essential role in the development of chromaffin cells [22], pituitary endocrine cells [9], pancreatic islets [7], and lung cancer cells of NE origin [23,24]. INSM1 promotes the trans-differentiation of pancreatic ductal and acinar cells into endocrine islets [25,26]. INSM1 is also required for the early differentiation of sympathetic neurons [22], sensory neurons of the dorsal root ganglion [27], and olfactory neurons [28]. *Insm1*-null mutant mouse demonstrates that Insm1 acts as a crucial component in the network that controls the differentiation of SA lineage. The sympathetic neurons of *Insm1*-deficient mice appear to differentiate correctly but with a delay, and their proliferation reduced. In contrast, the terminal differentiation of chromaffin cells is significantly affected by the *Insm1*-null mutation. The fetal lethality of *Insm1*-mutant mice is caused by a catecholamine deficiency, which is the specific marker of SA lineage, supporting the importance of *Insm1* in the development of SA lineage [22]. There is a wealth of phenotype similarities between *Mash-1* (*Ascl-1*) and *Insm1*-deficient mice including the delay of neuronal differentiation, reduced proliferation, and impaired differentiation of chromaffin cells, suggesting that *Insm1* might be the downstream target gene of *Mash-1,* whereas *Mash-1* activates endogenous Insm1 during the differentiation of chromaffin cells [22].

### 1.3. Neuroblastoma—A Neural Crest Derived Embryonal Malignancy

NB arises from SA progenitor cells within the NC that differentiate into sympathetic ganglion cells and adrenal catecholamine-secreting chromaffin cells [29,30,31]. NB is a malignancy of the sympathetic nervous system where the majority are located in the abdomen along the sympathetic chain and in the adrenal gland medulla area. NB tumors occurring in the early childhood represent 6–10% of pediatric tumors. NB accounts for 12–15% of all childhood cancer death and is the most common and deadly extracranial cancer [29,32,33]. The median age at diagnosis for NB is 17–18 months and approximately 40% of the patients are younger than 1 year at diagnosis, whereas less than 5% of the patients are older than 10 years [34].

**Figure 2:**
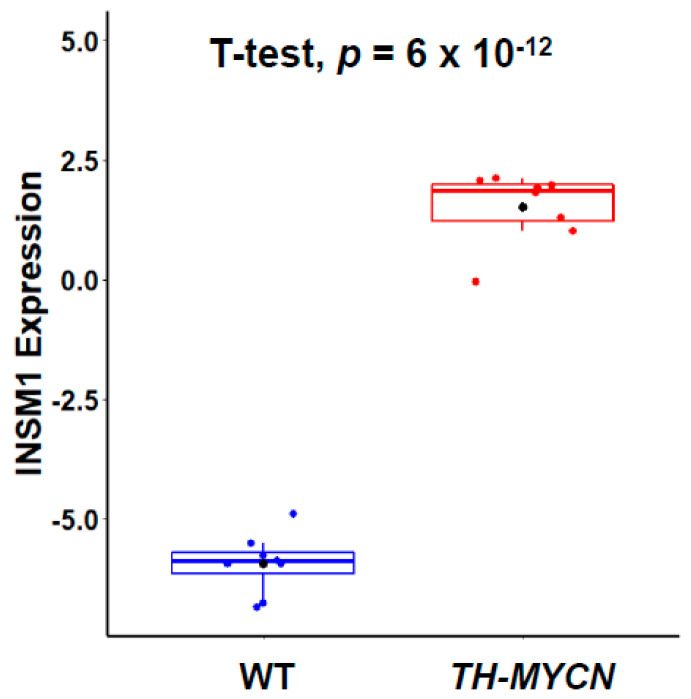
Insm1 expression in *TH-MYCN* transgenic mice. Insm1 expression in the abdominal superior mesenteric ganglion (SMG) dissected from 3-week-old TH-MYCN^+/−^ and WT mice. The boxplot shows the distribution and mean of Insm1 mRNA level with *t* test *p* = 6 × 10^−12^. The expression profiling data was obtained from GSE87784 dataset.

A *TH-MYCN* transgenic mouse model was generated using a tyrosine hydroxylase (TH)-promoter-driven human *MYCN* cDNA [35]. The *TH-MYCN* transgenic model spontaneously developed NB, which is morphologically and phenotypically similar to human high-risk NB [36]. This murine NB model mirrors human NB in many aspects of tumor formation including tumor locations, spinal cord involvement, histological presentations, cellular synapses, granule formation, and gains and losses of syntenic regions of chromosomes. The *TH-MYCN* mouse tumor is strongly positive for the expression of Insm1 (Figure 2). NB development in this mouse model is transgene dose-dependent. The homozygous mice develop NB tumor masses faster than hemizygous, at 4.0–6.9 weeks of age vs. 5.6–19 weeks of age. One-hundred percent of homozygous *TH-MYCN* mice have tumor growth from six weeks of age, but only fifty percent of hemizygous mice have tumor growth. Similar results were observed in another transgenic mouse model with Cre-conditional induction of *MYCN* in dopamine β-hydroxylase-expressing cells termed *LSL-MYCN/Dbh-iCre*, where clinically relevant NB tumor development occurred in 75% of mice [37]. The transduction of primary NC cells with over-expression levels of N-Myc back into the mice resulted in NB tumor formation [3,38]. Similar results were shown in the zebrafish model; ectopic expressions of N-Myc in neural crest cells induce NB development [39]. Taken together, these data strongly suggest that the over-expression of N-Myc in NC cells is sufficient for NB development and indicate that N-Myc is an important oncogenic driver in NB. Both clinical and experimental studies suggest that NB originates from the dysregulation of cellular processes during neural crest development [40,41,42].

### 1.4. N-Myc as an Oncogenic Driver in NB

A number of biological and genetic markers of NB have been investigated for the diagnosis, prognosis, and monitoring the treatment effects in NB patients. The *MYCN* status has been proven as the most significant marker for NB. It was strongly correlated with an advanced stage of disease and poor prognosis [30]. N-Myc expression is important for the regulation of NC cell migration and expansion during normal murine sympathoadrenal development. A high level of N-Myc expression is detected in the NC cells, which migrate into the dorsal aorta area. In normal embryonic development, the expression of N-Myc is gradually decreased during the differentiation of sympathetic neurons. The dysregulation of the N-Myc onco-protein plays a strong causative role in NB that induces widespread proliferation and inhibits the SA lineage cell apoptosis [43,44,45]. The molecular mechanisms of *MYCN* dysregulation are mostly driven by the disruption of transcription, translation, protein stability, apoptotic resistance, and metabolic flexibility [46,47,48].

Sympathoadrenal precursors that failed to differentiate into neural or chromaffin cells might end up developing NB [49]. The over-expression of N-Myc in sympathoadrenal precursors of zebrafish models not only abolishes the differentiation of chromaffin cells but also induces the development of NB [39]. N-Myc plays a crucial transcription factor in the regulation of cell proliferation and apoptosis. Persistent N-Myc expression during the maturation of SA precursor cells can cause the blockage of apoptosis and can induce abnormal proliferation of NB [35,50].

### 1.5. The Interaction between INSM1 and N-Myc

**Figure 3:**
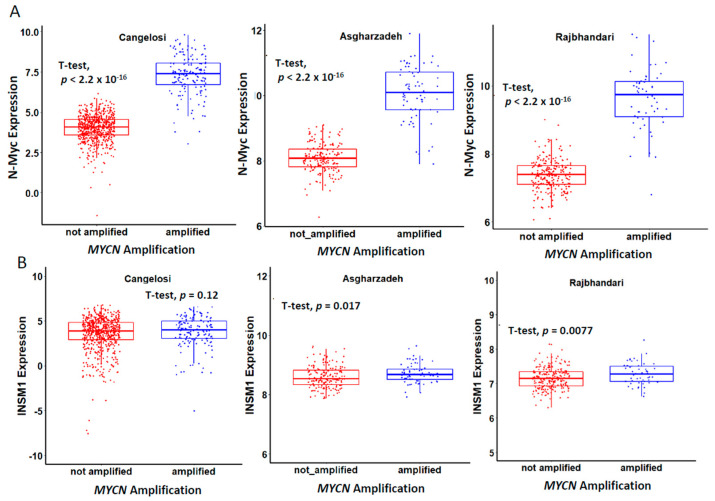
Correlation of N-Myc/INSM1 expression with *MYCN* gene amplification. (**A**) N-Myc expression positively correlates with *MYCN* amplification in NE patients derived from three different public cohorts, Cangelosi (782 samples), Asgharzadeh (247 samples) and Rajbhandari (273 samples). (**B**) INSM1 expression significantly correlates with *MYCN* amplification in NB patients derived from Asgharzadeh (247 samples), and Rajbhandari (273samples) but non-significant in Cangelosi (782samples) cohort.

**Figure 4:**
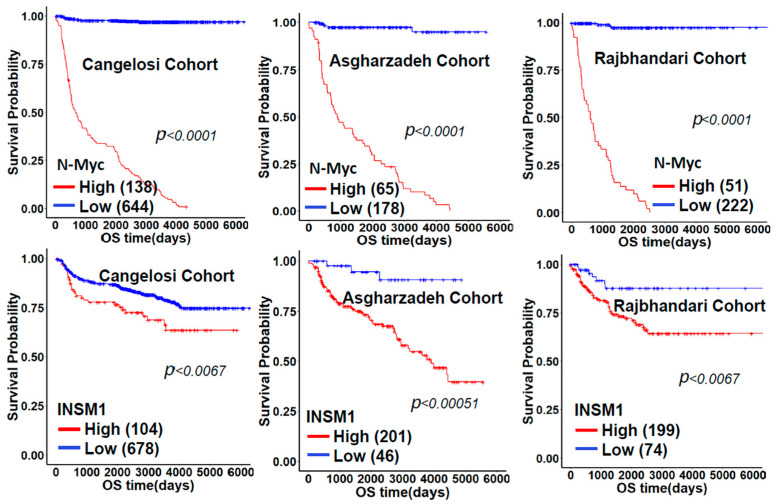
N-Mvc and INSM1 expression consistently associated with NB overall survival (OS). A Kaplan-Meier plot shows the OS time and survival probability in NB patients. NB patients derived from Cangelosi, Asgharzadeh, and Rajbhandari cohorts were divided into high-or low-N-Myc or INSM1 expression based on the cut-point using maximally selected rank statistics (maxstat).

Recently, the development of high-throughput technology investigating genomic DNA, RNA, and epigenetic profiles of clinical patient samples provided valuable information for analyzing the specific genes in the progression and prognosis of NB. We analyzed the correlation of the expression levels of N-Myc and INSM1 from three public NB datasets derived from GEO. *MYCN* amplification was identified as the first genetic biomarker of any cancer, specifically marking high-risk NB [51]. *MYCN* amplification is a well-confirmed prognostic factor that patients with *MYCN* amplification have poor prognoses and shorter overall survival time (OS). We analyzed the correlated expression level of N-Myc and *MYCN* amplification from three public NB datasets derived from GEO. The bioinformatics analysis indicates that patients with *MYCN* amplification as high-risk NB also have significantly high levels of N-Myc expression. However, INSM1 expression correlates with *MYCN* amplified versus non-amplified is less significant than N-Myc expression, which shows *p* values of 0.12, 0.017, or 0.0077, respectively (Figure 3). Furthermore, *MYCN* non-amplified NB tumors may have high c-Myc expression, which could still positively regulate INSM1 [46]. For the OS time analysis, the patients with a higher expression of INSM1 or N-Myc had OS times that were significantly shorter than those with lower expression levels (Figure 4, red line). Taken together, the clinical pathological result and bioinformatics analysis show that INSM1 is a strong diagnostic and prognostic biomarker for the evaluation of NB progression. The INSM1/N-Myc expression shows high clinical relevance in NB.

INSM1 can be considered an onco-fetal differentiation factor according to its elusive expression pattern in the fetal stage of NE precursors, silenced in adult normal tissues, and re-activated in tumors of NE origin. The re-expression of INSM1 in NB tumor cells contributes to the aggressive phenotype of a certain subtype of NB with enhanced N-Myc protein expression that is a well-confirmed oncogenic driver for the development of NB. Moreover, there is a feed-forward loop between INSM1 and N-Myc, N-Myc targets the *INSM1* gene via direct binding to the *INSM1* proximal promoter *E2-box* area and induces INSM1 expression. Additionally, INSM1 stabilizes the N-Myc protein via AKT phosphorylation and prevents the N-Myc protein from going into ubiquitination degradation [3,52]. The ectopic expression of INSM1 stimulates NB tumor growth in contrast to the knockdown of INSM1 that inhibits NB cell proliferation [53].

### 1.6. INSM1, a Novel Diagnostic and Prognostic Marker for NB

INSM1 functions as a transcription factor in NE differentiation. Recently, INSM1 has emerged in clinical practice as a sensitive and specific diagnostic biomarker in NE tumors (NETs). This novel biomarker provides pathologists a useful tool to distinguish NETs from non-NETs, and it represents a more sensitive NE marker as compared to the traditional markers, such as synaptophysin, chromogranin A, and CD56 [54]. INSM-1 nuclear expression in cytology specimens successfully shows that it is a robust NE marker. Numerous studies confirmed that INSM1 is a reliable immune-histochemical staining for NETs with high sensitivity and specificity [55,56,57].

NB is an NET that arises from the neural crest. The immune-histochemical profile of INSM1 in a cohort of peripheral neuroblastic tumors, using both tissue microarrays and whole-slide histologic sections, shows INSM1-positive staining in 78% of peripheral neuroblastic tumors and that no INSM1 signal was detected on the non-neuroblastic tumor [58]. Additional *INSM1* mRNA expression levels in a range of neurological cancers with NE features and other non-NE cancers were analyzed from the available datasets in an R2 Genomics Analysis and Visualization Platform (http//r2.amc.nl, accessed on 21 February 2022, Figure 5). The result shows that INSM1 has a high specificity and expression level in NB (red bar).

### 1.7. Application of INSM1 in Treatment of NB

The current treatment protocol for patients with high-risk NB remains limited, and the treated patients frequently developed chemo-resistant relapse after high-intensity conventional multimodal therapy [59]. Therefore, novel treatment protocols are needed [60]. Since a high percentage of the high-risk NB patients contain *MYCN* amplifications, targeting the *MYCN* signaling pathway could be beneficial for NB therapy [61,62]. We and others have shown that N-Myc is an oncogenic factor promoting NB progression and that increased N-Myc protein stability is crucial for NB cell growth by multiple feed-forward expression loops involving N-Myc trans-activation and repression of target genes [3,63,64]. Although *MYCN* amplification is a well-confirmed poor prognostic factor in NB, *MYCN* amplification is usually considered “undruggable” in standard care for *MYCN*-amplified high-risk NB patients. It will be a difficult strategy to design approaches for directly targeting or modifying the amplified *MYCN* gene. Therefore, we aim to target a co-factor of N-Myc in aggressive NB. We have provided evidence that INSM1 can increase the N-Myc protein stability and subsequently stimulate NB cell proliferation and anti-apoptosis. Additionally, N-Myc directly binds to the *INSM1* proximal promoter to activate endogenous INSM1 expression [3]. It is logical to use an *INSM1* promoter-driven luciferase assay for identifying novel inhibitors that could inhibit *INSM1* promoter activity [2]. We have identified several small molecules, such as 5′-iodotubercidin, plicamycin, romidepsin, panobinostat, and homoharringtonine targeting *INSM1* promoter activity, which exhibited potent inhibition of INSM1 and N-Myc protein expression as well as NB tumor cell growth [2,53]. Further evaluation of novel small molecules targeting aggressive NB should provide more insights into advancing NB therapeutic options.

## 2. Discussion

Multiple immune-histochemical studies demonstrated that the detection of INSM1 expression is a super sensitive and specific diagnostic biomarker in NETs as compared with conventional NE markers (CGA, SYP, and CD56) [54,55]. Functional studies of INSM1 in NETs revealed that INSM1 exhibited regulatory roles in the cellular signaling axis critical for cell growth and oncogenesis including NB [3,53]. *MYCN* amplification plays a particularly important role in the transformation of NB, representing about one- third of NB cases that belong to high-risk groups with higher mortality. However, *MYCN* amplification derives from the presence of multiple copies of the *MYCN* gene, which poses certain difficulties while direct targeting these multi-copy genes. Therefore, it is logical to design an alternative strategy targeting other co-factors associated with *MYCN* gene regulation. We have identified a novel NET biomarker, INSM1, which exhibits a feed-forward loop of regulating N-Myc protein stability [3]. Both gene expression levels are clinically associated with OS time as higher expression levels of N-Myc or INSM1 present shorter OS time. The clinical relevance of INSM1 and N-Myc expression in NB suggests that either molecule could be a prominent target for the suppression of an NB tumor.

An *INSM1* promoter-driven luciferase assay was developed [2]. We performed it on a small library screen using an *INSM1*-luciferase assay as a platform in NB. The assay result provides a high Z’ factor value suggesting its effectiveness in a chemical library screen. Several chemical compounds were identified as potent inhibitors for INSM1 and N-Myc expressions, as well as readily suppressing NB tumor cell growth. Interestingly, all of these small molecules possess a common feature of modifying INSM1 promoter regulation. Potentially, interference of the N-Myc activation of an INSM1 expression mediated through binding to the *E2-box* of *INSM1* proximal promoter could play a role in INSM1 regulation. These small molecules comprise HDAC inhibitors, natural compounds, and cellular metabolites that significantly reduce INSM1 activity [2,53]. The HDAC inhibitors were known to have pro-differentiation and apoptotic effects on the treatment of cancer including high-risk NB [65,66,67]. Although the HDAC inhibitor showed promise in NB treatment, it usually has a limited effect as a single agent due to the induction of drug resistance. Therefore, it is plausible to add other anti-NB small molecule compounds as combinational therapy. This approach should enhance the potency of treatment effects, lower the dosage of cytotoxicity, and reduce the possibility of drug resistance. Our preliminary data show that a combination of a natural compound (homoharringtonine) and an AKT1 inhibitor (A674563) that is important for N-Myc protein stability significantly reduces the expression of INSM1, N-Myc, and NB tumor cell growth (unpublished results). Combination therapy has been a hallmark of successful cancer treatment. Our *INSM1* promoter-driven luciferase screening-platform will be a valuable approach to screening additional novel small-molecules for NB cancer therapy. 

## Figures and Tables

**Figure 5:**
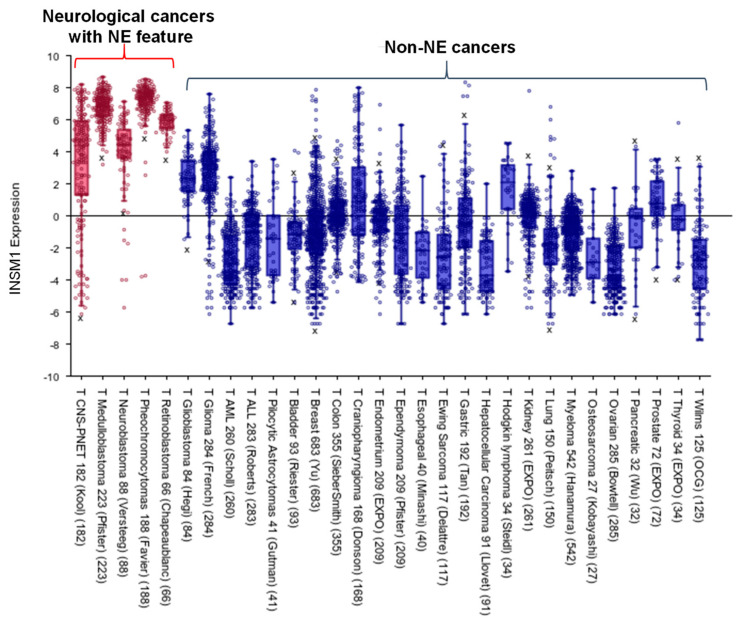
INSM1 expression in different tumors. INSM1 expression level in various tumors was obtained from publicly available datasets in R2: Genomics Analysis and Visualization Platform (http//r2.amc.nl, accessed on 21 February 2022). Analysis of INSM1 expression in neurological cancers with NE feature (red) and the other non-NE caners (blue) was across platforms with hs, u133p2, MAS5.0 data type for bioinformatics analyses.

## Data Availability

Not applicable.
